# The Action “Wanted!”: The Concept of Valency during the Early Steps of Mastering Chemistry

**DOI:** 10.11621/pir.2022.0404

**Published:** 2022-12-30

**Authors:** Elena V. Vysotskaya, Anastasia D. Lobanova, Maria A. Yanishevskaya

**Affiliations:** a Psychological Institute of Russian Academy of Education, Moscow, Russia

**Keywords:** Step-by-step concept formation, Activity approach, materialized form of action, interiorization, orientation

## Abstract

**Background:**

We apply the theory of step-by-step concept formation (Galperin) and the theory of learning activity (Davydov) to the practice of education and teacher training.

**Objective:**

This paper describes a feasible way to teach the basics of Galperin’s theory to students studying pedagogical psychology, by involving them in an exemplary educational module on combining chemical formulas according to the elements’ valency values.

**Design:**

We suggested that our students participate in an educational module which was designed as an example of how to materialize orientation components of action as the basis of concept formation. The “practical” action for mastering the valency concept was to combine the correct formula for a pair of elements, whose valency was provided, and correct the formulas made by someone else. However, the core “orientation” required an extended procedure of building a “molecule” structure with a special construction kit. The key challenge for the students was to coordinate their calculation of the number of bonds needed for the molecule, and name the exact total before they would receive their atom-tokens for constructing the model.

**Results:**

Our workshop participants took on the role of students facing their first encounter with chemistry, and embarked on the formation sequence. At the same time they analyzed the mistakes they had made by ignoring some procedural steps. Considered through the lens of Galperin’s theory, these “adult” mistakes proved how vital his theoretical principles are for educational design.

**Conclusion:**

Our workshop thus illustrated that the search for the proper action for concept formation within Galperin’s theory framework is a challenging task. The difficulties that our participants experienced while they worked as pupils revealed the divergence of didactic approaches. The effectiveness of the concept formation approach, even within our small exemplary educational module, once again confirmed the practical value of pedagogical psychology in general, and Galperin’s theory in particular.

## Introduction

The activity-based reconsideration of the development of psychological processes which Galperin conducted, brought about a fundamental shift in how to teach children to think conceptually. This course of action was supported by a number of pedagogical and psychological studies conducted within the Activity approach framework (Galperin & Talyzina 1957; Obukhova, 1968; Nikola, & Talyzina, 1972; Galperin, & Georgiev, 1969; Aidarova, 1978). Unfortunately, modern studies which rely on Galperin’s theory as their framework are rare (worth mentioning: Burmenskaya, & Evdokimova, 2007; Vysotskaya, & Rekhtman, 2012; Proyanenkova, 2014; Maksymenko, 2018). Meanwhile, current popular studies in education and the psychology for teaching are following and introducing methods based on misconceptions which were already overturned by Galperin (see the analysis in Arievitch, 2020). It is thus an urgent problem to transfer the means and meanings of Galperin’s theory to students, specifically future specialists in pedagogical psychology (Shabelnikov, 2017; Engeness, 2021).

In this paper we use our educational module on the valency concept, which was developed based on Galperin’s theory, in order to illustrate the major challenge of delivering concepts through students’ own actions; that challenge is the choice of a proper action.

At the core of assimilating concepts at school, according to Galperin (1957), are special activities and their intrinsic actions, which must include the concepts’ content as their absorbed “orientation” part, which are then turned into mental actions. In order to master the whole system of “landmarks” and operations which a concept bears and transmits over generations, one has to perform the actions according to the concept’s functionality. The materialized, external form of the action which “lies behind” the concept is thus placed (by Galperin) at the center of studies on the formation of concepts at school.

One of the first “projects” to examine the actions’ components and content, was a study on the formation of basic geometrical concepts through their defining characteristics (Galperin & Talyzina, 1957). The concepts’ definitions, which are usually provided in school, were revised to become the actual means of the students’ work. In this study the assimilation of the “action-mediated” way of orientation was introduced, as opposed to the “trial and error” search for one’s own way of handling some matter, a method which often leaves students ignorant about “what were all these definitions about.” The definitions of concepts were presented to students in their functional and operational aspects, so that the characteristics of geometrical objects from the definitions would “serve primarily to identify whether the given phenomenon belongs to the domain of the concept of interest, or not” within the special action of “recognition” (Galperin & Talyzina, 1957, p. 30). The concept-mediated action of recognition of geometrical figures was internalized and allowed students to “see” the objects in their “conceptual” aspect, while they dealt with them in school math problems.

Careful analysis of the actions of students who were making mistakes, led to closer examination of the deficiency of relying on the traditional object-oriented school definitions (Obukhova, 1968; Nikola, & Talyzina, 1972; Galperin, & Georgiev, 1969). The examination of the process of problem-solving highlighted the concepts’ “instrumental” role and the necessity for a special modelling space to support the materialized form of the desired action. These studies showed that the characteristics provided by the definitions were not sufficient for the orientation process required for the solution. Not only the objects which the concept can be applied to, but also the characteristics of the situation which demands a person act “conceptually,” vary from one problem to another.

Galperin wrote: “If a separate object is the bearer of the concept, the recognition by the characteristics goes smoothly; but if a set of objects appear to bear the concept, then the distinguishing features cannot point out how many of them there should be and why only together they present the object the concept is applied to” (Galperin, 1998, p. 305). The “concept-action” bond and the orientation functions of a concept within the process of concept formation thus demanded further examination within Galperin’s tradition of research (Davydov, 2008; Davydov’s modern followers — see Coles, 2021).

The special features of the “conceptual” form of action were described by Davydov (1960); the modelling scaffolds a shift from routine manipulation with objects towards the content of the concept: the product and means of theoretical thinking. Here the educational and pure cognitive meanings are opposed to the common-sense ones: modelling is aimed at purposes other than getting immediate pragmatic results.

In her experimental teaching of Russian morphology, Aidarova presented the qualitative leap from the object’s natural characteristics towards the conceptual meanings achieved through modelling (Aidarova, 1978). In this example the lexical meanings of the words interfered with their grammatical features, and the special modelling means, which reflected the morpheme structure of words, were introduced to separate and bind them conceptually. The work of building up the message, which required word-modification, repeatedly made students appeal to these artificial models. Further studies showed that special investigation of the components of the “materialized” form of action and the development of the proper models are necessary each time we need to scaffold the desired concept formation through appropriate action.

## Educational module on valency

In this paper we present an example of introducing special modelling means to support the materialized form of action. Our analysis of students’ and teachers’ difficulties also showed that without an adequate educational design, the traditional way of teaching appeals to formal procedures, which allow students to acquire the solution directly without “bothersome” modelling.

The concept of valency which we built our example upon, is introduced in the very beginning of learning chemistry, as students first encounter different formulas of substances. Students are to compose some compound formulas themselves based on the valency values of a given pair of elements, so that their formulas would match those written in the chemical books. Another task is to identify the valency of one element by its compound formula with another element, whose valency is provided.

What “landmarks” can the student refer to when he is performing the corresponding action?

The formulas themselves (elements’ symbols and indices) imply and demonstrate some regularity in the numbers placed near the symbols. A usual method used by teachers to explain how students should compose formulas themselves, is the “transposition” of the valency values to the indices of the elements in a “crisscross” manner (Winston, 1921). The action dictated by this instruction has some “conceptual” limitations, however: such operations may or may not lead to the right answer. Mistakes which children make when they try to combine the formulas themselves and confuse indices and valency values, are well-known. Those students who are good at math manage to overcome the mismatch with the formulas in the books by reducing the indices by common denominator, which is also how the majority of chemistry teachers instruct students to correct such mistakes.

We question the ability of the valency definitions contained in the textbooks to guide students’ own actions to compose a formula. Most descriptions in the basic chemistry textbooks are more or less like the following: valency is the capacity of an element’s atoms to combine with a certain number of other atoms. Valency is then usually illustrated through binding atoms of the element in question with univalent atoms (hydrogen, for example). Students successfully identify elements’ valency from these examples and combine formulas, but fail to deal with pairs of elements whose valency is other than one. The number of bonds of each atom in a compound is often used to illustrate valency: one can derive the valency values from a ready-made graphical representation of atoms with bars in between, but the number of bonds is not used by students to compose formulas according to the elements’ valency.

Counting the least common multiple of two valency values may help only in cases where students have mastered the ways to find the least common multiple as their active mental tool, which they can use fluently. When that is not the case, many students substitute finding the least common multiple by simple multiplication or even addition. As students proceed through the chemistry course, they adopt “roundabout” ways to deal with valency (the “crisscross method”), and diligent, hardworking students try to remember the most relevant formulas.

As we consider the ability to assimilate the valency concept as a psychological problem, and search for psychological tools to help overcome or even prevent students’ difficulties in such problem-solving, we have to elaborate the matter of their orientation in detail. The research questions here are 1) what are the components, means, and content of the action(s) at the core of assimilating the valency concept, and how is the formation of the orientation part of action to be scaffolded; and 2) what are the materialized forms of action at the early steps of concept formation and the procedure to develop it into the corresponding mental form, which will allow students to write the correct formula directly by the valency of the elements and recognize the valency “behind” new formulas.

“Logical-genetical” analysis (Davydov, 2008) aims to reconstruct the essential “conceptual” relationship of the matter of interest within an external form; it corresponds to Galperin’s call for the materialized form of action. However, the external form of action should not be understood as manipulating objects in order to motivate students and/or to grant them the right answers; and the modelling means of these actions does not serve as vivid illustrations to the ready-made formulas. In this respect, studies which suggest, for example, using Lego bricks to explain valency (Ruddick & Parrill, 2012), or the modern kits for chemistry classes with molecule models, do not represent an example of “materialized” form of action corresponding to concept formation. According to our analysis, the special action (orientational in its meaning, external and modelling in its form, and theoretical in its content) appropriate for valency concept acquisition demands the explication of the magnitude, which corresponds to the elements’ valences and makes tangible the meaning of the common multiple — the total number of bonds in a molecule. Students are to identify its value, which is disguised in a premade formula, and use it to assemble a “molecule” of the compound.

For such an educational design we needed to operationalize the definition of valency and find an appropriate model material to support the procedure, which would place the substantial relationship of the objects at the center of students’ consideration as they searched for the landmarks of their own action. The operationalized definition of valency should be one which implies and refers to the action of a student working with the compound’s formula. A formula contains element symbols and indices; where is the valency then? What do we do with the valency value in order to acquire the correct formula? Thus, we have chosen the following available definition, which is sufficient for building up the model (pen-and-paper) prototype of a substance formula:

Valency of an element signifies the number of bonds, through which each atom of an element is combined with atoms of another element^1^.

The special challenge was to make the students’ work with the model material purposeful and meaningful: to choose a problem which would develop into their own inquiry. Thus, we chose the construction kit with atom-units and bonds to fasten them, to be our model material (a “ball-and-stick model”). The kit’s “terms of use” demand that 1) students work together in pairs (each in charge for his own element units) and that 2) partners coordinate their calculations in order to acquire the exact total number of bonds needed to build the entire molecule prototype; then the teacher will hand out the fastening pieces (sticks). Only after that can the students have their atom-tokens.

**Figure 1.**
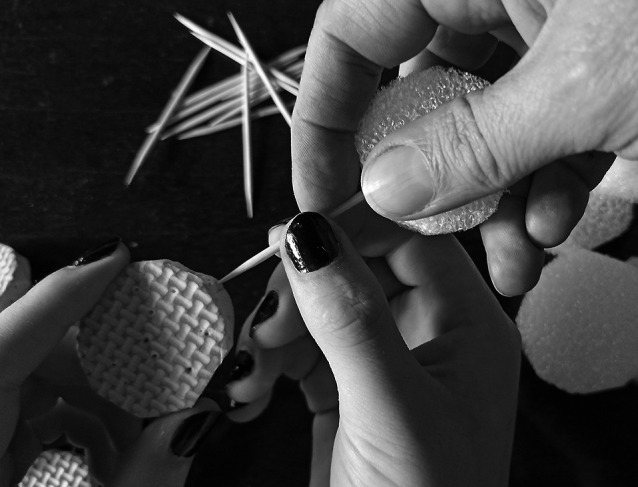
The construction kit: fastening two atoms together by the bonding “stick”

Each bond has to be used to connect two atoms of different elements (*Fig. 1*), while each partner is maintaining the proper valency of his own tokens. The preliminary calculation and selection of the number of fastening bonds, which has to be coordinated by both students in a pair, sets the learning task in our experimental teaching. The thoughtless manipulation of construction kit pieces (in case they are handed out simultaneously), without adjusting “partial” actions between partners will not allow students to properly build up the correct structures.

The search for the least common number of bonds in the compound of two elements for which the valency is given, thus comprises the content of the valency concept, and allows the student to develop the action towards building more complicated compounds later on.

## Results

We conducted a special teaching workshop to involve students of the pedagogical psychology department in the educational design of school subjects based on Galperin’s theoretical principles. We used materials from this workshop to illustrate the desired progression of the valency concept formation and the possible difficulties along the way: students’ common mistaken strategies, and teachers’ and educational designers’ prejudices.

There were 20 participants at our workshop — 15 students from Moscow State University and 5 guest students from other universities’ psychological departments. The students’ ages varied from 19 to 27 years; there was only one male student and 19 females (this gender ratio is typical for psychology departments).

Our workshop participants took on three roles at the same time: that of an educational designer, a would-be teacher, and a school student. After a short introduction on the basic idea and principles of Galperin’s theory, a “lesson” was conducted so that the participants would undertake the same steps of forming the valency concept themselves.

**Figure 2.**
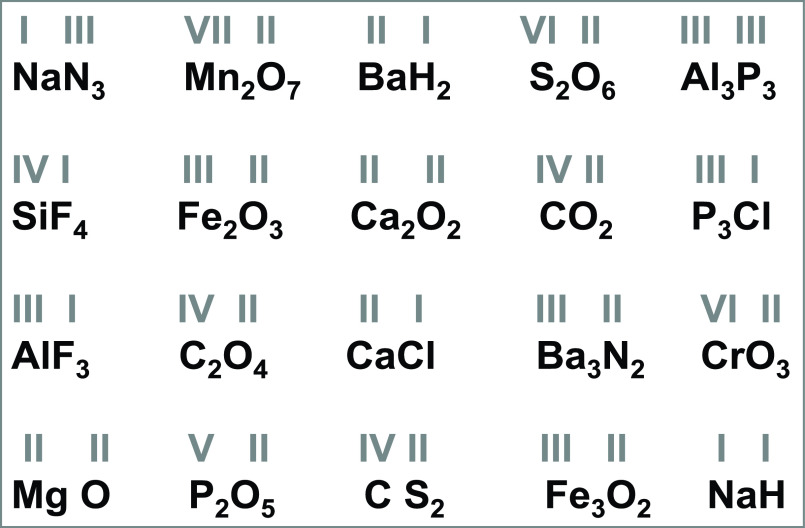
Introductory test. Check out the formulas, written by some students according to the elements’ valency (roman numerals above elements’ symbols)

First, a short oral test was given to assess the students’ understanding (*Fig. 2*). For school students the “prelude” would be, for example, to look through the textbook and see for themselves that there is an abundance of formulas consisting of symbols and indices. To understand them and compose the right ones themselves is what they should set out to learn.

**Figure 3.**
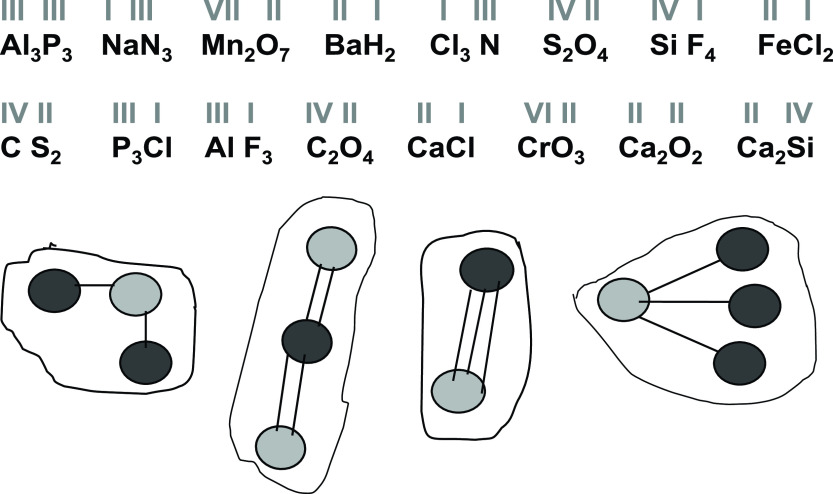
Note the formulas in the list which match the representations of “molecules.” Some incorrect formulas were presented on purpose to prompt a discussion

After the definition of valency was introduced, we checked whether the students grasped what the valency value signified in terms of bonds; students were to match the representations of “molecules” and the formulas (both correct and incorrect with respect to the elements’ valency) (*Fig. 3*).

After that, the teacher showed the students the construction kit and set the basic rule: the “bonds” were to be received before the “atoms”: students were to work together to choose the exact number of the bonds needed, and only then would the teacher hand them the “sticks.”

An example of coordinated work was then provided for a pair of elements; the students discussed the exact number of bonds needed for the phosphorus (P — III) and sulphur (S — II) compound, following the template provided:

“P” — student: In this substance there are three-valent atoms of phosphorus. Each atom of phosphorus makes three bonds. In a molecule with these atoms there can be three bonds, if there is one atom of phosphorus; six bonds, if there are two atoms; nine bonds, if there are three atoms; etc.

“S” —- student: In this substance there are two-valent atoms of sulphur. Each atom of sulphur makes two bonds. In a molecule with these atoms there can be two bonds, if there is one atom of sulphur; four bonds, if there are two atoms; six bonds, if there are three atoms; etc.

Then the key question was raised: “How many bonds do we take for this molecule? On what number will we agree? What number of bonds is the least to suit both partners?”

In the example provided it was six bonds.

Our participants now received a pair of elements and were to identify the number of bonds they would take from the teacher for their own molecule. The teacher handed out the bonds, after each pair repeated their argumentation following the template.

Then, each student in each pair was to decide how many atoms of his own elements make up this “molecule.” In the example with phosphorus (III) and sulphur (II), the reasoning went as follows:

“P” — student: Since the total number of bonds is six, I will need two atoms of phosphorus, because each token will have to be fastened with three bonds.

“S” — student: As the total number of bonds is six, I will need three atoms of sulphur, because each token will have to be fastened with two bonds.

**Figure 4.**
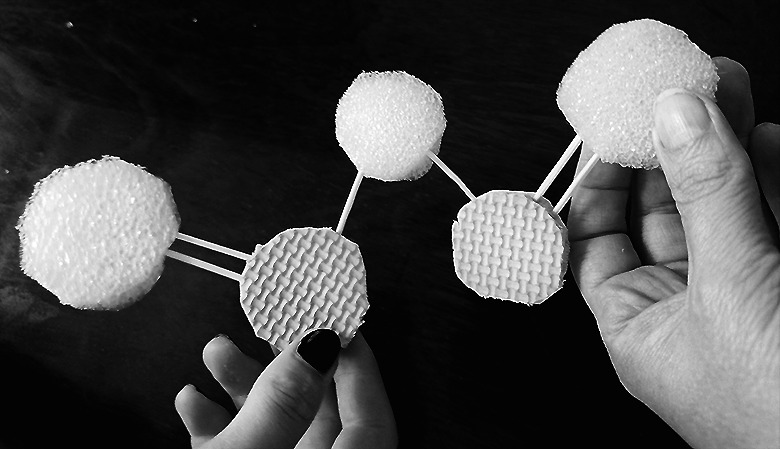
The P2S3 “molecule” model

Students took the tokens — each of his own element — and built the “molecule” (*Fig. 4*).

Finally, the formula of the compound was to be written: P_2_S_3_ in the example.

Then we laid out a series of tasks which scaffold the transition of students’ action through external articulated form, then “speaking to oneself,” and finally to the mental form of action (Galperin, 1998). Students composed the formulas for a pair of elements without using the construction pieces, but following the same template of oral reasoning: they were to coordinate their possible sets of bonds and come up with the proper total number of bonds for the compound, to identify the number of atoms for each element, and then to compose the formula.

The tasks of the last stages of interiorization were: 1) to write down the number of bonds and compose the formula; 2) to find the wrong formulas among the list and correct them; and 3) to fill in the indices for the formulas.

## Discussion

Our workshop was held as a demonstration rather than a real “full size” formation experiment. Nevertheless, we observed the typical mistakes that our previous experience showed that pupils make, as well as observed some interesting “grown-up” mistakes. These gave us a hint about the difficulties that psychology students experience when learning Galperin’s theory.

The common mistakes which students make derive from the empirical “criss-cross” strategy which they often come up with, since the indices do coincide with the valency values of the opposite symbols in some formulas. To reveal this kind of mistake, we placed a number of pitfalls in the introductory test, which demanded that students check out whether the formulas were composed according to the elements’ valency or not:

- wrong formulas which look right, because the indices coincide with the valency value above the symbols (*^III^ Ba_3_^II^ N_2_*)
- wrong formulas which look right, because the indices coincide with the valency value above the symbol across (*^VI^ S_2_^II^ O_6_*)
- correct formulas that look wrong, because their indices do not coincide with the valency values (*^IV^ C O^II^_2_* ; *Mg ^II II^ O*)

The results showed that the majority of the students fell into two of the three pitfalls: they pointed to formulas such as C_2_O_4_ as correct ones, and MgO they considered to be wrong, since their indices did not coincide with the valency values above. The discussion showed that students stuck with the “crisscross” strategy. They confessed their confusion with the CO_2_ formula, since they knew it was correct, but the numbers did not match.

**Figure 5.**
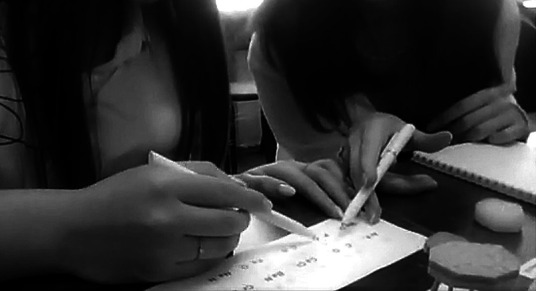

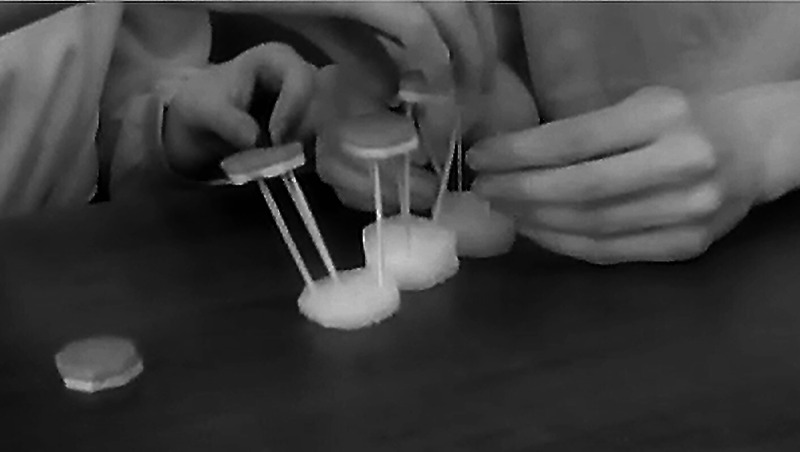
Building a proper model after each partner has chosen his atom-tokens corresponding to the total number of bonds chosen. Writing down the correct formula

**Figure 6.**
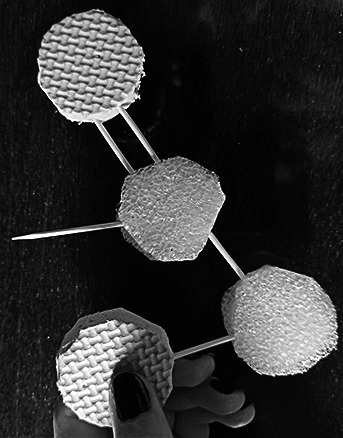

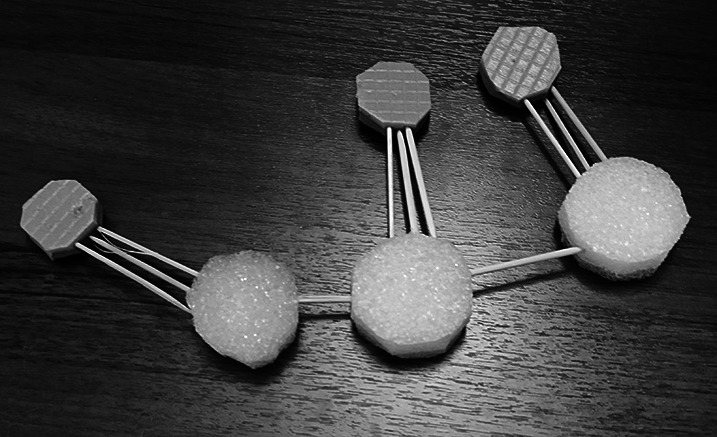

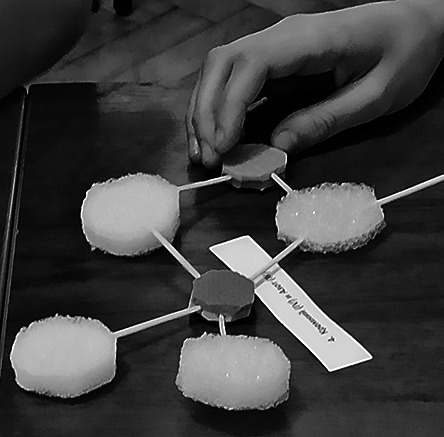
Building invalid constructions with the bonds and atoms

Those participants who followed the formation procedure with the construction kit step-by-step successfully combined the molecule and wrote down the formula (*Fig. 5*). There were some pairs of students who ignored the instruction and proceeded to fasten the molecule as soon as they received the bonding sticks (*Fig. 6*). They could not make a proper molecule: the valency values of their atoms were wrong, or the structure had “loose ends.” These mistakes are typical of school students when they start to make or draw graphic representations of molecule structures later on in a regular chemistry curriculum.

In the sequence of tasks that followed, many students tried to skip the “bothersome” extended step-by-step procedure of choosing the number of bonds, and then the number of each elements’ atoms corresponding to the total number of bonds. Eventually, they made mistakes. Most typically, they did not choose the smallest common number of bonds: for example, for Sulphur (VI) and Oxygen (II) they chose 12 bonds. Then the lecturer asked them to follow the template: to repeat what number of bonds there could be for each element and name the suitable total number of bonds. When the students, though reluctantly, performed the detailed procedure, they corrected their mistakes themselves.

Some students also failed to write the proper formula, since they did not articulate the choice of the number of atoms for each element in pairs, corresponding to the number of bonds. For example, after having chosen 12 bonds for Aluminum (III) and Carbon (IV), students wrote down the formula: Al_3_C_4_. Presumably, they skipped articulating that each atom of aluminum has three bonds and thus for 12 bonds, one needs four atoms of aluminum, three bonds each (and vice versa for carbon). Again, when asked to follow the procedure precisely, students could correct themselves.

The latter mistakes are of a special kind: they happen due to taking “shortcuts” in procedure and are corrected by including the missing steps. Students, especially adult ones who are used to performing operations mentally and individually, try to optimize the “cumbersome” procedure and miss the essential “coordination” steps, which are of the utmost importance here. The essential content of the valency concept is exactly the way the valency values of the two elements are to be coordinated through the total number of bonds. If the substantial basis for this coordination is not assimilated by students, then within their individual formulas composition they will have to conduct some “roundabout” ways to produce the indices.

These mistakes do not happen outside the formation procedure, and they are an important indicator that students do not accept the necessity of doing the task in the required way, which they find cumbersome and excessively extended, although it assuredly leads to correct formulas. Thus, students ignore the orientation functions of the means provided and miss their opportunity to acquire the concept. A feasible explanation of the origin of these mistakes is the discrepancy between the new step-by-step formation procedure and the students’ own learning experience within the traditional paradigm. However, the usual way of explaining the topic with simplistic demonstrations shows only the execution part of action and keeps the orientation part out of sight.

The analyses of the origin of these mistakes through the lens of Galperin’s theory allowed the workshop participants to understand the difference between the naïve and conceptual comprehension of students’ difficulties, and the meaning of the fundamental elaboration of the orientation procedure, which mediates the correct executive part of action.

## Conclusion

Our major result was the design of a feasible way of introducing students to Galperin’s theory through their participation in real concept-formation, where each component of the step-by-step formation procedure can be observed. Here the materialized form of action deserves the utmost attention, as it may be mistaken for mere visual illustrations and hands-on manipulations by those who are not familiar with Galperin’s theory. Thus, with this article we tried to clarify the difference and the originality of his activity approach to formation of a concept.

The central content of the valency concept is the substantial coordination of students’ partial actions within shared choice of the total number of bonds, which is at the core of the orientation process. The transition from the joint form of action to the individual one comprises the most important part of the interiorization and has to be scaffolded with the appropriate procedure.

As students considered their mistakes through the lens of “orientation development,” the basic principles of Galperin’s theory proved to be vital. It was an opportunity to direct students’ attention to the content of the action “behind” a concept, especially its orientation part, as its quality defines the flawless performance of a practical action on the first try. The simple demonstrations of visual “aids” whose role is limited to the illustrative, deprives students of the conceptual basis for their own orientation. The choice of the proper action and the design of its materialized form are thus still a challenge, for which Galperin’s theory provides essential guidelines for school education innovations.

## Limitations

The purposes of our research demand from our future teaching series a more detailed collection and description of the students’ actions for each step of the learning trajectory for a broader sample. This might allow its statistical processing and the definition of the factors leading to successful or poor performance of the critical tasks.

## Footnotes

1 We do not go in detail of the modern notion of different types of bonds (covalent, ionic, metall) here, as it is more object-related and cannot scaffold students’ actions at the very first encounter with chemical formulas (Nelson, 2002; Brown, 2013; Sindhu, 2021 and other modern descriptions of the nature of chemical bonding for teachers and students).
